# Editorial: Assessment and monitoring of human movement

**DOI:** 10.3389/fphys.2025.1686977

**Published:** 2025-08-29

**Authors:** Cristian Romagnoli, Vincenzo Bonaiuto, Elvira Padua, Giuseppe Annino

**Affiliations:** ^1^ Department of Human Science and Promotion of Quality of Life, Human Performance, Sport Training, Health Education Laboratory, San Raffaele University, Rome, Italy; ^2^ Department of Industrial Engineering, Sports Engineering Laboratory, University of Rome Tor Vergata, Rome, Italy; ^3^ Department of Medicine Systems, Human Performance Laboratory, Centre of Space Bio-Medicine, University of Rome Tor Vergata, Rome, Italy

**Keywords:** functional assessment, sports biomechanics, sports physiology, artificial intelligence (AI), physical activity, human movement analysis, wearable sensors

The study of human movement is a well-established multidisciplinary research field that draws expertise from biomechanics, functional anatomy, physiology, and neuroscience, with an ever-increasing emphasis on technology and engineering (Verrelli et al., 2021a; 2023; Xu et al.; Edriss et al., 2024b; Gao et al.; Jiang et al.; Jiang et al.; Khan et al.; Miao et al.; Zhang et al.; Zhang et al.; Zhou et al.; Zhu et al.; Romagnoli et al., 2025b). The demand for accurate and reliable tools to assess physical performance continues to rise, particularly in sports science and clinical practice (Chen and Shen, 2017; Fan et al., 2019; Annino et al., 2021; Verrelli et al., 2021b; Zanela et al., 2022; Yang et al.; Zając et al.). Traditionally, functional assessments were confined to controlled environments, where gold-standard technologies such as motion capture systems, force platforms, and metabolic analyzers could guarantee precision (Cavagna, 1975; Luhtanen and Komi, 1978; Ehara et al., 1997; Hausswirth et al., 2007; Lucía et al., 2008). However, despite their accuracy, these tools often lacked ecological validity: performance observed under laboratory conditions did not reflect the biomechanical aspect of movement and, in particular, lost the specificity of sports performance (Sale and MacDougall, 1981). Bridging this gap has become a central challenge in sports science. For this reason, sport engineering has allowed us to bring the laboratory assessment directly to the race/training field (Bosco et al., 1995; Cormie et al., 2007; Bonaiuto et al., 2020; Romagnoli et al.; Goreham and Ladouceur). The miniaturization and increased affordability of sensors, coupled with advances in wireless data transmission and computational power, have paved the way for a new generation of wearable, non-invasive devices (Chambers et al., 2015; Aroganam et al., 2019; Xu et al.; Edriss et al., 2024b; Xiang et al.; Yang). These tools can now measure a range of biomechanical, physiological, and kinematic variables such as acceleration, angular velocity, muscle activity, joint angles, heart rate, and more during actual sporting performances or daily activities (Hausswirth et al., 2007; Giggins et al., 2022; Bonfiglio et al.; Hermosilla Perona et al., 2024; Papini et al.; Ren et al.; Romagnoli et al.; Caprioli et al., 2025). The impact of this transition is profound: by enabling the monitoring of athletes, patients (Alahmari and Reddy; Herrera-Valenzuela et al.; Kang et al.; Liu and Bai; Miyazaki et al.; Mo et al.), and healthy individuals in their natural contexts (Chen et al.; Guo et al.; Ko et al.; Liang; Xiang et al.), movement science is gaining unprecedented depth and relevance. In elite sports, the potential of such devices is well recognized. The ability to collect and analyze real-time data during training or competition allows coaches and practitioners to fine-tune performance variables with precision, in addition to monitoring fatigue and recovery (Taborri et al., 2020; Guppy et al., 2022; Daniel et al.). Whether tracking running load via GPS in soccer or analyzing stroke dynamics with inertial sensors in kayaking or swimming, the quantitative approach to sports performance is now a cornerstone of evidence-based practice (Romagnoli et al., 2022; Romagnoli et al.; Santos et al., 2022; Goreham and Ladouceur). Moreover, the integration of these tools with video analysis systems—particularly those enhanced by deep learning and 3D reconstruction-offers new possibilities for unobtrusive and highly detailed motion analysis, even in competitive scenarios (Annino et al., 2023; Blanco-Coloma et al.; Edriss et al., 2024a; 2025b; 2025a; Najlaoui et al., 2024; Romagnoli et al., 2025a). The clinical relevance of these technologies is equally promising. In rehabilitation and neurodegenerative disease management, wearable sensors are being used to assess gait patterns, balance, and motor coordination in patients with conditions such as Parkinson’s disease, multiple sclerosis, and Alzheimer’s disease (Das et al., 2022; Zhao et al., 2023). Functional metrics (e.g., symmetry, kinematic and dynamic parameters, EMG activity, harmony of motion, and temporal variability) are now being used as objective markers to guide and to help therapeutic decisions and track disease progression. Despite these advances, important challenges remain. One of the most pressing needs is the standardization and validation of sensor-based measures across populations, devices, and contexts. As the ecosystem of wearable technologies expands, ensuring data reliability and interoperability becomes essential for scientific progress and clinical translation. Equally important is the development of user-friendly interfaces that make advanced analytics accessible to non-expert users, including coaches, clinicians, and patients, without compromising data integrity or interpretability. A further frontier lies in the integration of multimodal data. Combining information from different sources, such as inertial sensors, electromyography, video, and physiological monitors, can provide a more holistic picture of motor function (Fan et al., 2019; Stetter et al., 2019; Zago et al., 2019; Meng et al., 2021; Zanela et al., 2022). The application of artificial intelligence and machine learning is proving valuable here, enabling the extraction of meaningful patterns from complex datasets and the construction of predictive models for performance outcomes, injury risk, and therapeutic response (Nasr et al., 2021; Ammar et al.; Bogaert et al.). This Research Topic [63 articles submitted, with 36 studies accepted (57%) and 27 rejected (43%)] reflects the vitality of the field and the growing interest in the functional assessment of human movement as a tool for both performance enhancement and health promotion. The contributions gathered here showcase methodological innovations, application-specific protocols, and novel devices tailored to diverse populations and environments. Together, they underscore a central message: the future of movement science lies in its ability to operate seamlessly across settings, to speak the language of multiple disciplines, and to generate knowledge that is not only reliable and valid but also actionable. In conclusion, the technological transformation of human movement analysis marks a turning point in how we understand, measure, and apply physical performance data. From elite athletes striving for excellence to patients seeking autonomy in daily life, the ability to assess movement with accuracy, in context, and over time is becoming a cornerstone of both sports science and healthcare. We hope that this collection of research contributions will foster new collaborations and inspire further innovations aimed at making movement analysis ever more accessible, meaningful, and impactful.
